# Effect of *Kelulut* Honey Nanoparticles Coating on the Changes of Respiration Rate, Ascorbic Acid, and Total Phenolic Content of Papaya (*Carica papaya* L.) during Cold Storage

**DOI:** 10.3390/foods10020432

**Published:** 2021-02-16

**Authors:** Bernard Maringgal, Norhashila Hashim, Intan Syafinaz Mohamed Amin Tawakkal, Mahmud Tengku Muda Mohamed, Muhammad Hazwan Hamzah, Maimunah Mohd Ali

**Affiliations:** 1 Department of Biological and Agricultural Engineering, Faculty of Engineering, Universiti Putra Malaysia, 43400 Serdang, Selangor, Malaysia; bernardmaringgal@yahoo.com (B.M.); hazwanhamzah@upm.edu.my (M.H.H.); maimunah_mohdali@ymail.com (M.M.A.); 2 Department of Agriculture Malaysia, Putrajaya 62624, Malaysia; 3 SMART Farming Technology Research Centre (SFTRC), Faculty of Engineering, Universiti Putra Malaysia, Serdang 43400, Selangor, Malaysia; 4 Department of Process and Food Engineering, Faculty of Engineering, Universiti Putra Malaysia, Serdang 43400, Selangor, Malaysia; intanamin@upm.edu.my; 5 Department of Crop Science, Faculty of Agriculture, Universiti Putra Malaysia, Serdang 43400, Selangor, Malaysia; mtmm@upm.edu.my

**Keywords:** kinetic model, Peleg constant, papaya, respiration rate, nanoparticles coating, shelf life

## Abstract

This study evaluated the respiration rate of coated and uncoated (control) papayas (*Carica papaya* L.) with 15% of *Kelulut* honey (KH) nanoparticles (Nps) coating solution during cold storage at 12 ± 1 °C for 21 days. The respiration rate of the papayas significantly changed during storage, with an increase in CO_2_ and a decrease in O_2_ and C_2_H_4_, while the ascorbic acid and total phenolic content was maintained. The changes in respiration rate were rather slower for coated papayas when compared to control ones. A kinetic model was established from the experimental data to describe the changes of O_2_, CO_2_, and C_2_H_4_ production in papayas throughout the storage period. All O_2_, CO_2_, and C_2_H_4_ were experimentally retrieved from a closed system method and then represented by the Peleg model. The outcomes indicated the Peleg constant *K*_1_ and *K*_2_, which were gained from linear regression analysis and coefficients of determination (*R*^2^), seemed to fit well with the experimental data, whereby the *R*^2^ values exceeded 0.85 for both coated and control papayas. The model confirmed both the capability and predictability aspects of the respiration rate displayed by papayas coated with KH Nps throughout the cold storage period. This is supported by the differences in the stomatal aperture of coated and control papaya shown by microstructural images.

## 1. Introduction

Papaya is a tropical climacteric fruit with high respiration rates and ethylene (C_2_H_4_) production during ripening. The fruit is rich in vitamins, minerals, and dietary antioxidants [1]. Nevertheless, it has a short life span due to its climacteric respiration pattern. Papaya of colour indices 2 and 3 can last between five and seven days at ambient temperature, while those with colour indices 4 and 5 can maintain their quality for only two to three days [2]. The short life span enhances the rate of natural deterioration, such as physicochemical damages that eventually increase its susceptibility to diseases and infection [3]. This results in post-harvest loss, a deficit in production yield, as well as a limitation to long-distance export destinations.

The two post-harvest handling strategies, namely modified atmosphere packaging (MAP) and controlled atmosphere (CA), can effectively control the quality of fresh produce by modifying the gas exchange of the fresh produce and its nearby atmosphere [4,5,6]. Edible coating, an effective MAP strategy, has displayed exceptional outcomes for maintaining the quality of tropical climacteric fruits such as papaya [5,7], mango [8,9], and banana [10,11]. According to Maringgal et al. [12], an edible coating is capable of creating a semipermeable barrier on fruit surfaces that can decrease the fruit respiration rate and C_2_H_4_ production and result in the maximum quality retention of fruit during storage. Several studies have reported that edible coating can increase the shelf life of papaya during storage by decreasing respiration rate, slowing down the senescence process, and maintaining the chemical compositions, such as ascorbic acid (AA) and total phenolic content (TPC) of papaya [13,14,15,16].

Fruit respiration rate can be described by its carbon dioxide (CO_2_) generation and oxygen (O_2_) consumption. Recently, Maringgal et al. [17] have suggested that the *Kelulut* honey (KH) coating can potentially minimise the papaya respiration rate during cold storage at 12 ± 1 °C and prolong their shelf life up to 12 days. The authors also added that KH coating can act as a physical barrier that leads to the rapid CO_2_ accumulation rate of the coated fruit. On the other hand, Nicolaï et al. [18] explained that CO_2_ may have some direct and indirect controlling effect on the respiration metabolism and C_2_H_4_ production. The respiration rate can be determined via a flow-through system or closed system [19]. For the flow-through system, the fruit sample is placed in a non-permeable container where a gas mixture flows at a constant rate. In the closed system, the fruit sample is filled with gas-tight containers of known volume which contain ambient air as the initial atmosphere.

Respiration rate modelling is an effective method to evaluate the respiratory kinetics of fresh produce. There are several respiration rate models that have been used in fruits, such as the Arrhenius, Michaelis–Menten, and Peleg models [17,20]. The Peleg model has been applied to analyse the experimental data on the changing of O_2_, CO_2_, and C_2_H_4_ concentrations as well as describe the rate of gas composition function. The suitability of the Peleg model in describing respiration rate has been acknowledged for some fruits, including apples [21,22], bananas [23,24], fresh-cut papaya [20], mangoes [25], and kiwiberry [26]. However, studies on using Peleg modelling to determine the impact of nano-coating on the rate of respiration for the whole papaya are almost non-existent. Most of the studies have focused more on its potential in enhancing the quality of the fruit [27,28,29]. For instance, Vieira et al. [30] examined the efficacy of hydroxypropyl methylcellulose coating incorporated with silver nanoparticles (Nps) on the post-harvest shelf life of papaya. It was found that nano-coating successfully maintained the quality of papayas, extended the shelf life of papaya up to 14 days, controlled the development of anthracnose disease, and reduced the rate of respiration of the fruit during storage. According to Maringgal et al. [31], this could be due to the reduced Nps size that leads to superior Nps penetration, absorption, as well as migration into fruits, thus promoting an effective coating system.

Therefore, this study aims to (1) identify the impact of nano-coating on papaya’s respiration rate, AA, as well as TPC, and (2) investigate the respiration rate kinetic model of nano-coated papaya using the Peleg model in order to describe the function of gas composition and storage day.

## 2. Materials and Methods

### 2.1. Bio-Synthesis of Kelulut Honey Nanoparticles

The KH Nps were generated as described by Maringgal et al. [31] through deposition as well as precipitation of calcium carbonate (CaCO_3_) in KH via biosynthesis.

### 2.2. Fruit Sample Preparation

A total of 30 fresh papayas (*Carica papaya* L. cv. Sekaki) at colour index 2 (green with traces of yellow) were employed for this research. The fruit were bought from a commercial wholesaler located in Selangor, Malaysia. The chosen papayas were ensured to be uniform in size and shape, with an average weight ranging between 1000 g and 1500 g. The chosen samples also did not have external injuries and pathogenic infection, and were given additional preventive treatment by immersing into 0.01% chlorinated water that was prepared from 5% sodium hypochlorite. The treatments were divided into two categories, namely control (uncoated) and coated with KH Nps at 15% concentration, with each category consisting of 15 fruit.

Nano-coating of KH Nps at 15% concentration (*w/v*) containing 1% glycerol and Tween 20 were prepared as described by Maringgal et al. [31]. The papayas were dipped into the KH Nps concentrations for 1 min and then air-dried for a duration of 2 h. The samples were packed in commercial corrugated boxes in a single layer and stored at 12 ± 1 °C with 85–90% relative humidity for 21 days. The observed changes in respiration rate, C_2_H_4_ production, AA, and TPC were recorded at seven-day intervals in which three samples were used for each analysis.

### 2.3. Determination of Gas Exchange and Ethylene (C_2_H_4_) Production

The papaya’s respiration rate was determined by performing methods detailed by Maringgal et al. [17] with minor modification. A closed system was adopted, whereby the fruit was enclosed for 5 h (previously 2 h) in an airtight container at room temperature. All samples were subjected to the measurement of internal gas concentration (O_2_, CO_2_, and C_2_H_4_), whereby 1 mL sample of internal gas from the central cavity of the papaya was drawn out thrice with the use of a syringe after being incubated for 5 h inside the airtight container. A gas chromatography instrument (Agilent 6890N Network, California, CA, USA) equipped with a stainless-steel column (Carboxen-1010 PLOT, 30 m × 0.53 mm I.D) and a thermal conductivity detector (TCD/methanizer-FID, 230 °C) was used, into which the gas samples were injected. The carrier gas, helium, was employed at 3.0 mL·min^−1^ flow rate. Before and after sample analyses, the peak areas that were identified for standard gas mixtures were measured. The measurements of the initial and final O_2_, CO_2_, and C_2_H_4_ were carried out and employed to compute the respiration rate. The measurements used the expression of nmol kg^−1^ s^−1^ for O_2_ and CO_2_, and μL/kg·h for C_2_H_4_.

### 2.4. Ascorbic Acid (AA) Assay

The AA was identified according to Wall [32], whereby 20 g of papaya sample was homogenised with 80 mL of 3% metaphosphoric acid (HPO_3_) by using a high-speed blender for a minute. The AA was calculated using Equation (1) and expressed in mg/100 g.

(1) $$AA=\frac{\mathrm{mL}\;dye\;used\;\times \;dye\;factor\;\times \;volume\;of\;product\;\left(100\;\mathrm{mL}\right)\;\times \;100}{Weight\;of\;sample\;\left(20\;g\right)\;\times \;volume\;of\;sample\;for\;titration\;\left(5\;\mathrm{mL}\right)}$$

### 2.5. Determination of the Total Phenolic Content (TPC)

The determination of the TPC was carried out by using the Folin–Ciocalteu reagent prescribed by Abu Bakar et al. [33] and modified by Mendy et al. [3]. First, 300 μL of papaya extract was mixed with 2250 μL of Folin–Ciocalteu reagent, vortexed for 15 s, and allowed to stand for 5 min at room temperature. Next, the mixture was added to 2250 μL of sodium bicarbonate solution (60 g/L). After that, the mixture was allowed to stand for 2 h (previously 90 min) in the dark at room temperature, whereby the absorbance was computed with the use of a Multiskan GO microplate spectrophotometer (Thermo Scientific 1510, Vantaa, Finland) at 750 nm. The outcomes were expressed as mg of gallic acid equivalent per 100 g for the fresh sample (mg GAE/100 g).

### 2.6. Microstructural Microscopy

For microstructure observation, the papaya peel cubes were fixed in a solution containing 4% glutaraldehyde for 2 days at 4 °C. With the use of 0.1 M sodium cacodylate buffer (pH 7.6), the peel cubes were rinsed three times for 30 min, one after another. In a solution containing 1% (*w/v*) osmium tetraoxide, the papaya peel cubes were post-fixed at 4 °C for 2 h. Using 0.1 M sodium cacodylate buffer solution (pH 7.6), every fixed papaya peel cube was again rinsed three times for 30 min. In a series of acetone concentrations ranging from 35% to 100%, the cubes were dehydrated during three changes of acetone solution. Next, a specimen basket was used to transfer the papaya peel cubes. It was then placed into a critical point dryer (LEICA EM CPD030, Wetzlar, Germany) for 30 min. The drying papaya peel cubes were mounted onto the stub using double-sided adhesive and gold-coated by a gold sputter coater. The samples were observed using a scanning electron microscope (JEOL JSM-IT100, InTouchScope™, Tokyo, Japan).

### 2.7. Data Analysis, Experimental Data Modelling, and Model Parameter Estimation

The two-way analysis of variance (ANOVA) was applied in determining the statistical variances among the papaya samples and storage days. The mean significant variances between the parameters were compared using the least significant difference (LSD) test at a *p* < 0.05 significance level. Statistical analyses were carried out with the use of SAS 9.4 software (Version 9.4, SAS Institute, Cary, NC, USA).

The experimental data of O_2_, CO_2_, and C_2_H_4_ were determined by measuring the gas O_2_, CO_2_, and C_2_H_4_ concentrations over the storage days. Changes observed in the evaluated gases over storage days were calculated by employing the closed system equations, as given in the following [23]:

(2) $$R_{O_{2}}=\;\frac{\left(y_{O}^{t^{i}}{}_{2}-y_{O}^{t^{f}}{}_{2}\right)\;\times \;V}{100\;\times \;M\;\times \;t}$$

(3) $$R_{CO_{2}}=\;\frac{\left(y_{CO}^{t^{i}}{}_{2}-y_{CO}^{t^{f}}{}_{2}\right)\;\times \;V}{100\;\times \;M\;\times \;t}$$

(4) $$R_{C_{2}H_{4}}=\;\frac{\left(y_{C_{2}H}^{t^{i}}{}_{4}-y_{C_{2}H}^{t^{f}}{}_{4}\right)\;\times \;V}{1000\;\times \;M\;\times \;t}$$

where $R_{O_{2}}$ is the O_2_ rate, nmol kg^−1^ s^−1^; $R_{CO_{2}}$ is the CO_2_ rate, nmol kg^−1^ s^−1^; $R_{C_{2}H_{4}}$ is the C_2_H_4_ rate, μL/kg·h; $\left(y_{O}^{t^{i}}{}_{2}-y_{O}^{t^{f}}{}_{2}\right)$ is the O_2_ concentration, %; $\left(y_{CO}^{t^{i}}{}_{2}-y_{CO}^{t^{f}}{}_{2}\right)$ is the CO_2_ concentration, %; $\left(y_{CH}^{t^{i}}{}_{4}-y_{CH}^{t^{f}}{}_{4}\right)$ is the C_2_H_4_ concentration, %; *V* is the volume of the incubation airtight container, m^3^; *M* is the mass of the fruit, kg; and *t* is the incubation time, h.

The respiratory quotient (RQ) was determined as the CO_2_ respiration rate divided by the O_2_ respiration rate. The data were plotted using a response surface analysis (Design Expert Software, Version 11, Stat-Ease Inc., Minneapolis, MN, USA).

## 3. Results and Discussion

### 3.1. Changes in Gas Concentrations

**Figure 1:**
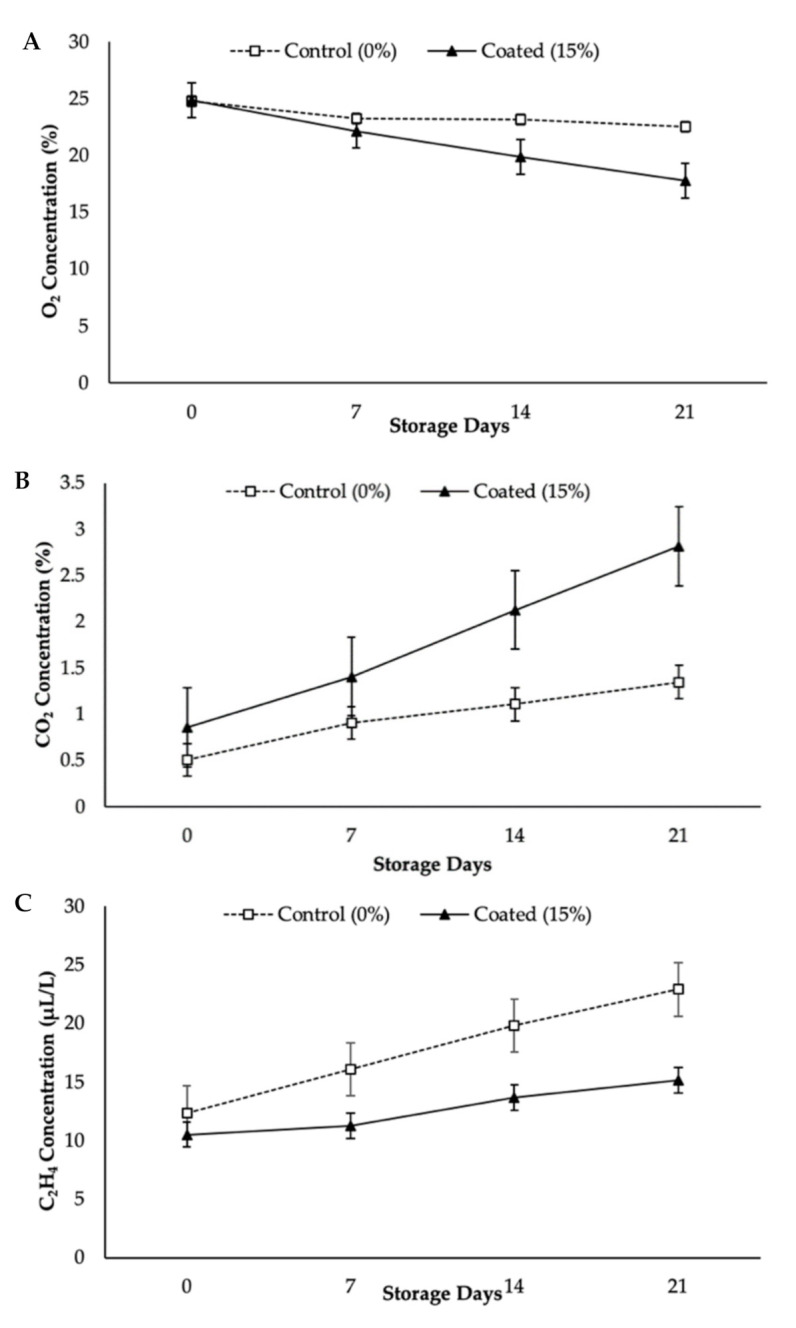
Changes in gas concentration in terms of (**A**) O_2_, (**B**) CO_2_, and (**C**) C_2_H_4_ at 0, 7, 14, and 21 days. Vertical bars indicated LSD (*p* < 0.05). LSD: least significant difference.

Table 1 shows the main and interaction effects of KH Nps coating and storage duration on the gas concentration, respiration rate, RQ, AA, and TPC of papaya fruits at 12 ± 1 °C. On the other hand, Table 2 presents a two-way ANOVA of KH Nps coating and storage durations on the experimental parameters of the papaya fruits. The ANOVA result showed the significant effect of the experimental factors (KH Nps coating and storage durations) on the experimental parameters at *p* < 0.05. Changes in the concentrations of O_2_, CO_2_, and C_2_H_4_ during the storage period at 12 ± 1 °C were observed, as illustrated in Figure 1.

The changes in gas concentrations of coated papayas were significantly (*p* < 0.05) delayed, when compared to the control ones. A gradual reduction in O_2_ concentration was noted for all the treatments throughout the 21 days (see Figure 1A). Nonetheless, escalating trends were observed for CO_2_ and C_2_H_4_ concentrations in both coated and control papayas (see Figure 1B,C). For coated papaya, the initial CO_2_ percentage increased from 0.86% to 2.82%, whereas the control papaya recorded an increase in CO_2_ from 0.51% to 1.35%. The production of C_2_H_4_ was higher on day 21, with the value of 22.92 µL/L and 15.19 µL/L for control and coated papayas, respectively. The increased CO_2_ concentration for KH Nps-coated papayas was in line with the reduction in O_2_ concentration during storage. Xu et al. [34] reported that the accumulation of CO_2_ in coated fruits was because of a reduced respiration rate that inhibited physiological effects towards the fruits. Meindrawan et al. [35] revealed that nanocomposite coating can decrease the amount of O_2_ for respiration and limit diffusion of CO_2_ out of the fruit tissues. The KH Nps coating might have restricted the gas exchange through papaya peel, thus retarding C_2_H_4_ production. The study outcomes are in agreement with prior observational studies, which reported that high internal CO_2_ concentration in fruit can hinder C_2_H_4_ generation, thus delaying the ripening of fruit [36].

### 3.2. Respiration Rate

**Figure 2:**
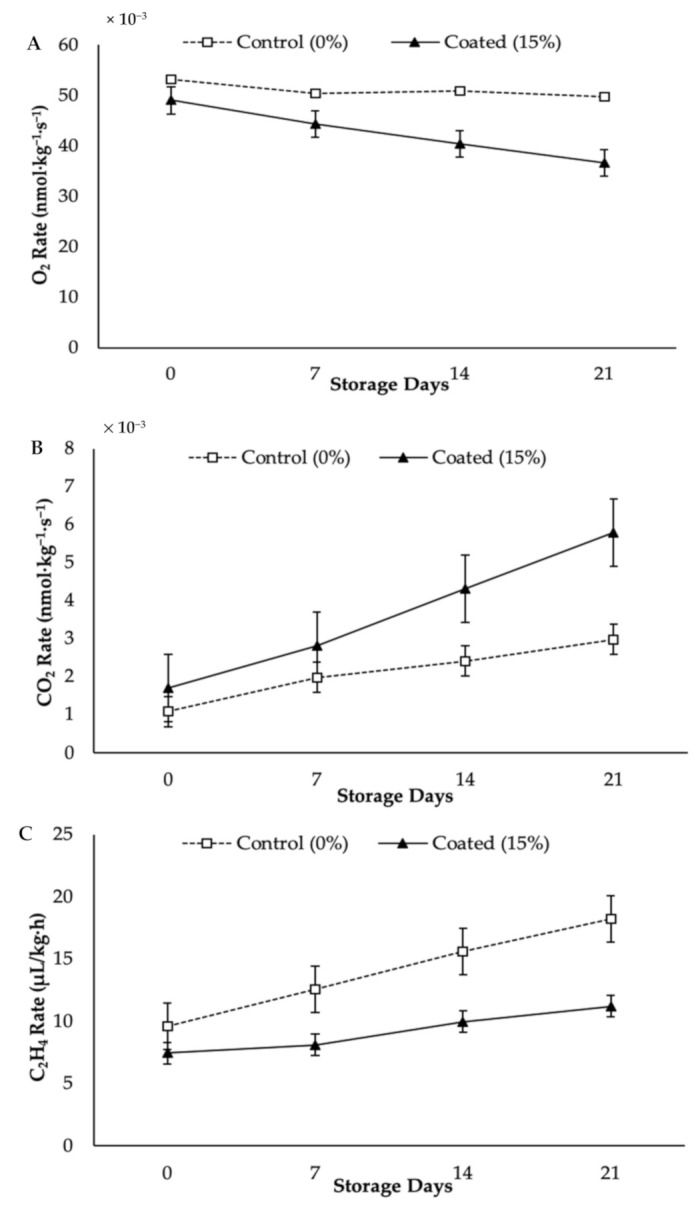
Respiration rate in terms of (**A**) O_2_, (**B**) CO_2_, (**C**) C_2_H_4_ production at 0, 7, 14, and 21 days. Vertical bars indicated LSD (*p* < 0.05).

Both respiration rate and C_2_H_4_ production rate in fruit are regarded as good indices to determine shelf life [37]. Figure 2 illustrates the significant decrease (*p* < 0.05) in respiration rate in the coated papaya but a gradual increase in respiration rate in the control papaya. This signifies that the O_2_ rate in the coated papaya was lower during cold storage when compared to the control samples (see Figure 2A). After 21 days of storage, the highest O_2_ rates were 49.78 × 10^−3^ nmol kg^−1^ s^−1^ and 36.66 × 10^−3^ nmol kg^−1^ s^−1^ for the control papaya and the coated papaya, respectively.

The effect of KH Nps coating on CO_2_ rate appeared to vary significantly (*p* < 0.05). The CO_2_ rate was found to increase in parallel with the storage period for both the control and the coated papayas (see Figure 2B). The coated papayas showed the highest CO_2_ rate, at 5.79 × 10^−3^ nmol kg^−1^ s^−1^ on day 21 from its initial rate at 1.71 × 10^−3^ nmol kg^−1^ s^−1^. Likewise, the control papayas displayed the lowest CO_2_ rate after 21 days of storage, at 2.98 × 10^−3^ nmol kg^−1^ s^−1^, whereas the initial CO_2_ rate was 1.09 × 10^−3^ nmol kg^−1^ s^−1^.

The C_2_H_4_ production rate differed significantly (*p* < 0.05) due to the KH Nps coating treatment (see Figure 2C). The C_2_H_4_ production rate in control fruit escalated rapidly and reached its peak after 21 days of storage at 18.21 μL/kg·h. On the contrary, the coated papaya exhibited the lowest value for C_2_H_4_ production rate after 21 days of storage (11.24 μL/kg·h) as well as a slow increase in C_2_H_4_ production rate upon completion of the storage period.

Such delays in respiration and C_2_H_4_ production rates for the coated papayas, in comparison to those of the control, may suggest that the KH Nps edible coating exerted an obstacle to gaseous exchange. This present study discovered that the KH Nps coating generated a modified atmosphere with high CO_2_ and low O_2_ in the papaya, which reduced both respiratory and C_2_H_4_ production rates. Past studies have shown that the reduced rates of respiration and C_2_H_4_ production in coated papaya resulted in delayed senescence [38]. Similarly, Mendy et al. [3] reported that papaya coated with *Aloe vera* gel coating experienced less weight loss during the storage period when compared to control, which was ascribed to the coatings having provided a semi-permeable layer against gas movement and a resulting decrease in respiration rate. The pattern of respiration and C_2_H_4_ production rates recorded in this study is in agreement with the findings reported by Ali et al. [13], whereby papaya coating with chitosan suppressed both respiration and C_2_H_4_ production rates through the modification of the fruit’s internal atmosphere.

Moreover, Maringgal et al. [17] found that the uncoated papaya displayed an early increase in respiration rate compared to papayas coated with 1.0% and 1.5% KH for 12 days of cold storage period. The reduction in respiration and C_2_H_4_ production rates due to coating treatment has been reported by many researchers for a range of fruits, including mango [39], mandarin [40], guava [41], and banana [11].

**Figure 3:**
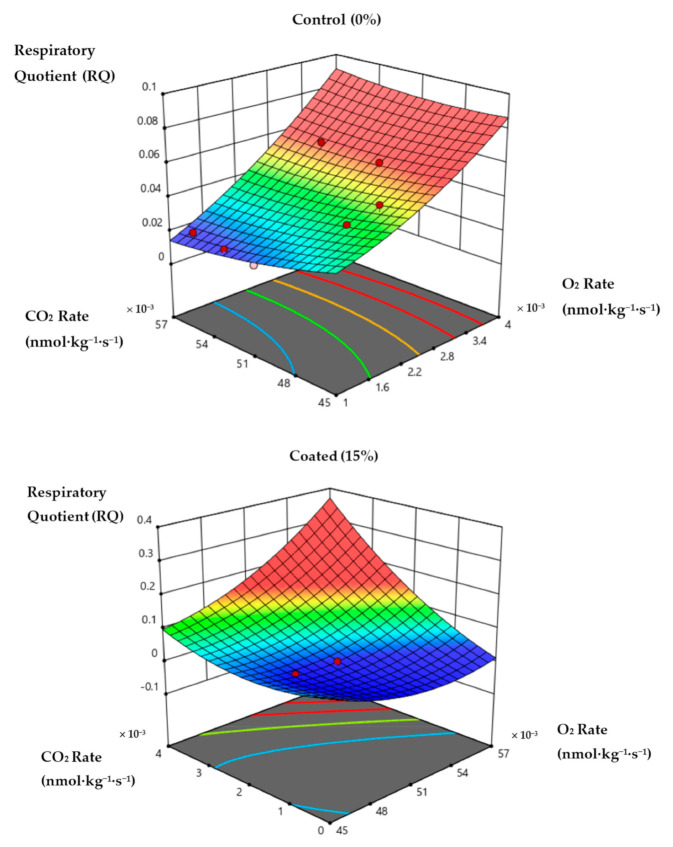
The effect of CO_2_ and O_2_ partial pressures on the RQ of control and coated papaya fruit at 12 ± 1 °C. The red dot symbols represent data points above and below the response surface, respectively (*p* < 0.05).

Additionally, Sapper et al. [42] described that the RQ was influenced by changes in respiration rate, indicating the existence of the substrate used during the respiration process of fruit. The RQ was relying on both O_2_ and CO_2_ production rates, resulting in a decrease in O_2_ and an increase in CO_2_ [43]. This study revealed the RQ exhibited a significant effect on the storage day and coating treatment of papaya (Table 1 and Table 2). The RQ gradually increased for both coated and control papaya during the 21 days of storage, as shown in Figure 3. However, it was noted that the RQ was higher for coated papaya as compared to control papaya. This suggested that the transition of metabolic substrates from carbohydrates to organic acids was higher in coated papaya, which was in agreement with the findings of Sapper et al. [42] and Fagundes et al. [44]. The authors found that an RQ equal to 1 signifies that the metabolic substrates are carbohydrates, while an RQ higher than 1 signifies that the metabolic substrates are organic acids.

### 3.3. Model Parameter Estimation

The Peleg equations (Equations (2)–(4)), which were modified to reflect curvilinear changes in terms of gas concentration over storage day, were employed to model the computed respiration rates. The inverse of change in gas concentration and the respiration rate models with storage period were plotted, whereby model parameters *K*_1_ and *K*_2_ were estimated via linear regression analysis. The value of *K*_1_ was estimated using the curve’s slope, whereas *K*_2_ is denoted by the intercept on the *y*-axis. Table 3 presents the values of *K*_1_ and *K*_2_, as well as the coefficient of determination (*R*^2^). The *R*^2^ seemed to be very high (*R*^2^ > 0.85), which signified an exceptional fit between the experimental data and the Peleg model for coating treatment.

Both *K*_1_ and *K*_2_ values increased for the model parameters of O_2_, which was influenced by the KH Nps coating treatment. For instance, the values of *K*_1_ and *K*_2_ for coated papaya were 2.35 and 27.06, respectively. Comparatively, the values of *K*_1_ and *K*_2_ for control papaya were 0.68 and 25.15, respectively. In this present study, *K*_2_ was more influenced by the coating treatment. A higher value of *K*_2_ was also reported by Rahman et al. [20] for fresh-cut papaya, Bhande et al. [23] for banana, as well as Mahajan and Goswami [21] for apples. The variance between *K*_1_ and *K*_2_ is attributable to both gaseous and physical changes of fruit during the storage period. The Peleg model described *K*_1_ as referring to the rate constant, and it is physically related to both the consumption as well as the evolution of gases starting from the very initial stage, while *K*_2_ refers to the model’s capacity constant, implying the respective gas content that can be attained by the system up to infinity. The *R*^2^ values in terms of O_2_ were high; 0.997 and 0.859 for coated and control papayas, respectively.

The *K*_2_ appeared to be lower than *K*_1_ for coated papaya in terms of CO_2_. The values of *K*_1_ and *K*_2_ for coated papaya were 0.66 and 0.16, respectively; while those for control papaya were 0.27 and 0.29, respectively. The *R*^2^ values in terms of CO_2_ were also high; 0.997 and 0.976 for coated and control papayas, respectively.

**Figure 4:**
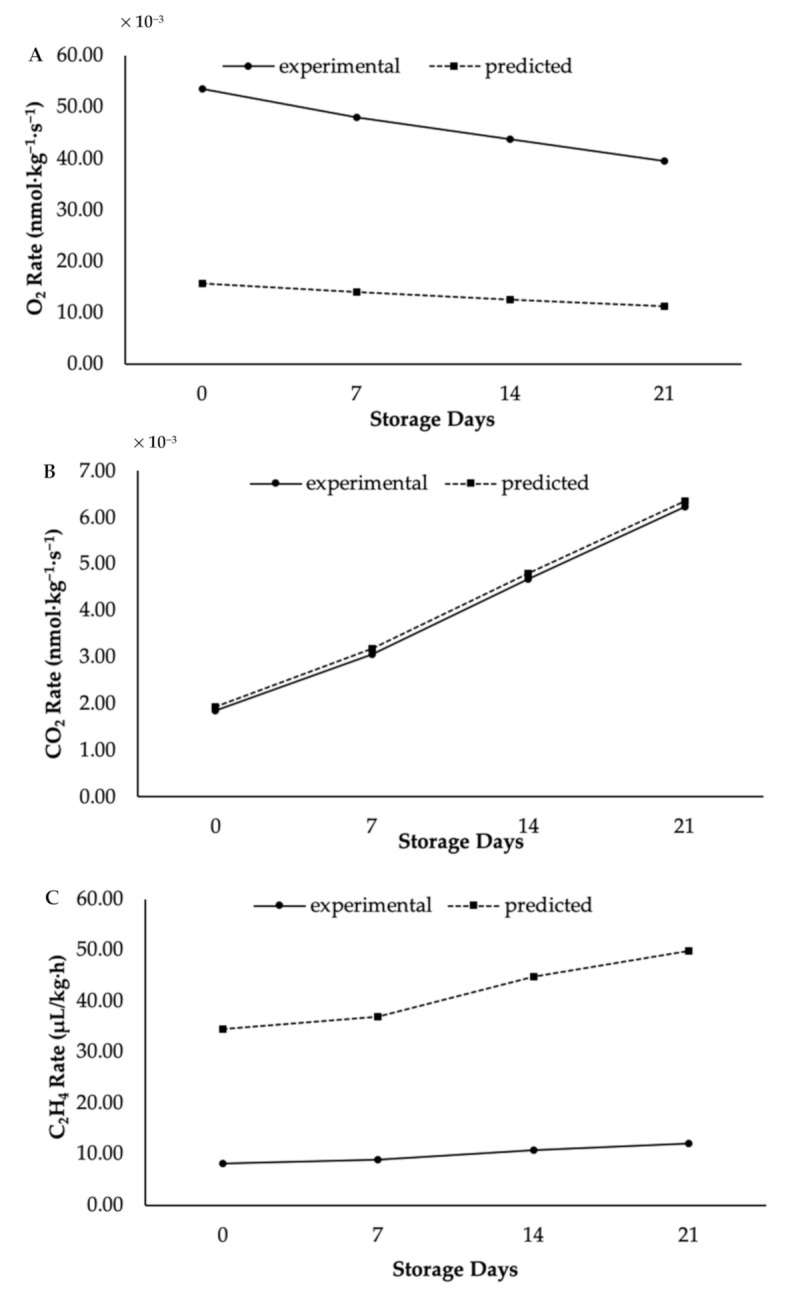
Experimental and predicted respiration rates in term of (**A**) O_2_, (**B**) CO_2_, and (**C**) C_2_H_4_ production in KH Nps-coated papaya.

**Figure 5:**
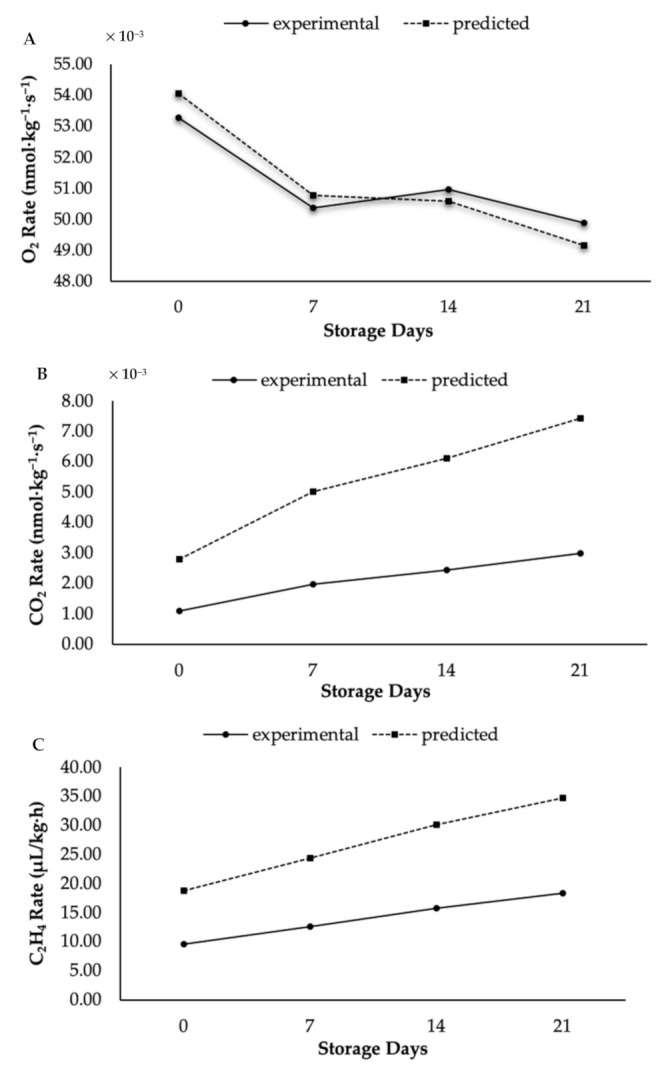
Experimental and predicted respiration rates in term of (**A**) O_2_, (**B**) CO_2_ and (**C**) C_2_H_4_ production in control papaya.

Next, the relationship between coating treatment and model parameters in terms of C_2_H_4_ concentration showed a similar trend to the O_2_ concentration. For instance, the values of *K*_1_ and *K*_2_ for coated papayas were 1.63 and 8.59, respectively; while those for control papayas were 3.53 and 9.01. The *R*^2^ values in terms of C_2_H_4_ were 0.967 for coated papayas and 0.998 for control papayas. Figure 4 and Figure 5 illustrate that most of the experimental values were lower than those predicted. The figures display the variances between experimental and predicted respiration rates for coated (see Figure 4) and control (see Figure 5) papayas in terms of O_2_, CO_2_, and C_2_H_4_ concentrations. Moreover, the empirical equation developed by Peleg [45] has proven its capability for respiration rate prediction in several fresh produce products [20]. Data developed by this equation plays a significant role in the design of successful modified atmospheric systems for nano-coated papaya. Based on the results of this study, both respiration and C_2_H_4_ production rates of the papaya were well described by the Peleg kinetic models, and their values have been shown to be significantly influenced by the coating treatments.

### 3.4. Microscopy Observation

**Figure 6:**
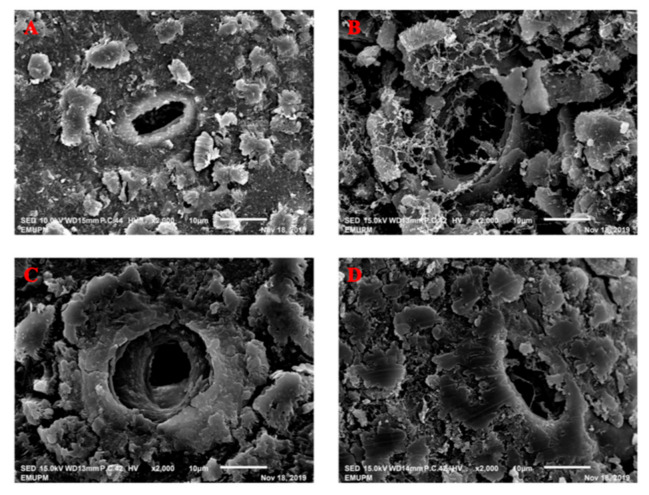
Microstructural images of stomatal aperture on papaya peels at different storage periods: (**A**) control at day 0, (**B**) coated at day 0, (**C**) control at day 21, and (**D**) coated at day 21.

The effect of KH Nps coating on the papaya’s surface characteristics was observed by using a scanning electron microscope (SEM). Figure 6A,B indicates the stomatal aperture on papaya peel in control and coated fruits on day 0 of storage. After 21 days of storage, KH Nps coating at 15% concentration continued to cover the stomata on the papaya peel (Figure 6D), hence causing a delay in the ripening process. The KH Nps coating might have protected the stomata on the papaya peels, thus decreasing the respiration rate. Nevertheless, the control papaya peel resulted in exposed stomatal aperture, as shown in Figure 6C, thus reflecting the increase in both respiration and C_2_H_4_ production rates, apart from affecting both the post-harvest qualities and physicochemical characteristics of papaya throughout the storage period.

These results were consistent with the research of Jongsri et al. [39], where it was observed that there was a low molecular weight of chitosan coating, which hence conferred protection and covered the stomata of mango peels. This contributes to the delay in the process of ripening during storage. Deng et al. [46] showed that the non-homogeneous coating present on the fruit’s surface has the potential to enhance the mass transfer across the fruit surface, thus increasing respiration rate as well as a fungal infection. In the current research, a low respiration rate and delayed C_2_H_4_ production were maintained by the coated papayas.

### 3.5. Effect on Ascorbic Acid (AA) and Total Phenolic Content (TPC)

**Figure 7:**
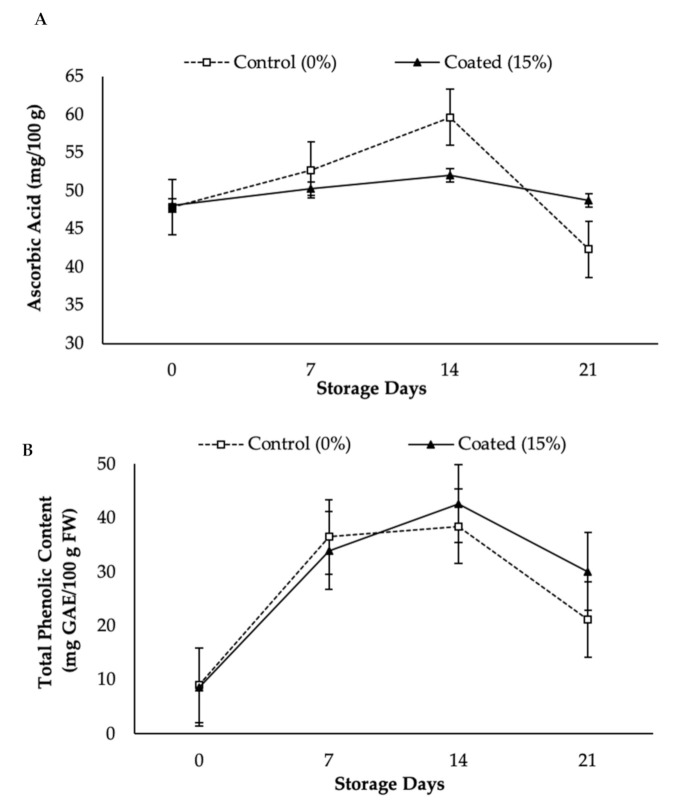
Effect of Ascorbic Acid (**A**) and Total Phenolic Content (**B**) in KH Nps coated papaya stored at 12 ± 1 °C for 21 storage days. Vertical bars indicated LSD (*p* < 0.05). GAE: gallic acid equivalent. FW: fresh weight.

AA, or vitamin C, is a water-soluble vitamin and is an important vitamin in papaya [47,48]. Lee and Kader [49] asserted that AA content can be easily degraded due to improper post-harvest handling and storage conditions. In this study, AA content was significantly (*p* < 0.05) greater for the control papayas than the coated papayas (see Figure 7A). The AA content increased during storage, and the highest AA was recorded for control papaya on day 14 at 59.65 mg/100 g, and 52.01 mg/100 g for coated papaya. This finding is supported by an early study performed by Wills and Widjanarko [50], who explained that AA content will increase in parallel with the ripening stages, then decline thereafter due to senescence.

Furthermore, KH Nps-coated papaya displayed a slower initial increase in AA as compared to the control papaya. At day 21, the AA content of KH Nps-coated papaya maintained a higher value (48.69 mg/100 g) compared to control papaya (42.33 mg/100 g). This suggests that the KH Nps coating slowed down the synthesis of AA during storage. This finding was consistent with Ali et al. [13], wherein the authors found that papaya coating with chitosan is able to reduce the respiration rate of fruit by increasing the CO_2_ concentration, thus slowing down the synthesis of AA during storage. The fruit were stored at 12 ± 1 °C with relative humidity (RH)of 85–90% for four weeks. Additionally, the higher AA retention by KH Nps coating treatment could be associated with the ability of the coating to act as a gas barrier to reduce the O_2_ tension in the papaya fruit tissue. This statement was supported by the study of Maftoonazad and Ramaswamy [51], who revealed that the atmosphere composition around the fruit has a significant role in AA retention during storage. Magwaza et al. [47] and Madani et al. [52] added that lower C_2_H_4_ production in fruits improved AA retention, which was confirmed when C_2_H_4_ production was measured and revealed a low concentration in KH Nps-coated papaya.

Phenolics are the secondary metabolites existent in plants that have antioxidant properties during the oxidative stress process [53]. It appears that the TPC of papaya varied significantly (*p* < 0.05) due to KH Nps coating (Figure 7B). The highest TPC (42.64 mg GAE/100 g fresh weight (FW)) was recorded for the coated papaya fruits on day 14. The TPC for the control papaya rapidly increased during the storage and reached the maximum (38.48 mg GAE/100 g FW) at day 14, however, it was unable to maintain the TPC rate until day 21 (21.20 mg GAE/100 g FW). A low value of TPC or a sharp reduction in TPC after 14 days in the control papaya could be due to the higher respiration rate, which may have resulted in the loss of TPC because of the degradation of specific phenolic compounds [37].

At the end of storage (day 21), the coated papaya was able to maintain the TPC better than control papaya and this may be because the KH Nps coating reduced the metabolism in the coated papaya. In other words, the KH Nps coating treatment has the ability to maintain the TPC of papaya fruit during cold storage. Similar contributions have been made by Ayón-Reyna et al. [54], who found that untreated papaya had a lower TPC as compared to papaya treated with hydrothermal-calcium chloride. This has also been explored in a prior study by Ghasemnezhad et al. [55], in which the authors revealed the decrease in TPC of coated apricot due to the deterioration process.

## 4. Conclusions

The kinetic change in respiration rate and the C_2_H_4_ production of control and coated papayas were studied in this research. The respiration rate and C_2_H_4_ production in papayas were significantly affected by the KH Nps coating. The current study also showed that KH Nps coating can be used as a conserving material, extending the shelf life by inhibiting the respiration rate and C_2_H_4_ production, while maintaining the AA and TPC, in papaya. Furthermore, differences in stomatal aperture were observed in coated and control papaya after 21 days of cold storage. This study also suggested that respiration data generated by a closed system could be employed to model the respiration rates. The respiration rate and C_2_H_4_ production that were predicted by the Peleg model obtained *R*^2^ values higher than 0.85 for both coated and control papaya. This showed that KH Nps is a promising edible coating that can be employed in commercial post-harvest applications for extending the shelf life of papayas.

## Figures and Tables

**Table 1:** Main and interaction effects of *Kelulut* honey (KH) nanoparticles (Nps) coating and storage durations (0, 7, 14, and 21 days) on the gas concentration, respiration rate, respiratory quotient (RQ), ascorbic acid (AA), and total phenolic content (TPC) of papaya fruits at 12 ± 1 °C.

Factors% KH Nps Coating (C)	Gas Concentration			Respiration Rate			RQ	AA (mg/100 g)	TPC (mg GAE/100 g FW)
	O_2_ %	CO_2_%	C_2_H_4_μL/L	O_2_nmol kg^−1^ s^−1^	CO_2_nmol kg^−1^ s^−1^	C_2_H_4_μL/kg·h			
0—Control	23.44 A	0.97 B	17.82 B	51.07 × 10^−3^ A	2.12 × 10^−3^ B	14.01 B	0.040 B	53.72 A	26.28 B
15—Coated	21.19 B	1.81 A	12.67 A	42.5 × 10^−3^ B	3.66 × 10^−3^ A	9.21 A	0.087 A	49.63 B	28.65 A
Storage duration (days), (SD)									
0	24.83 A	0.68 D	11.47 D	51.13 × 10^−3^ A	1.40 × 10^−3^ D	8.54 D	0.03 D	47.95 D	8.82 D
7	22.73 B	1.16 C	13.70 C	47.39 × 10^−3^ B	2.39 × 10^−3^ C	10.35 C	0.05 C	49. 96 C	34.95 B
14	21.56 C	1.62 B	16.76 B	45.58 × 10^−3^ BC	3.37 × 10^−3^ B	12.81 B	0.08 B	52.96 B	40.32 A
21	20.17 D	2.08 A	19.05 A	43.20 × 10^−3^ C	4.39 × 10^−3^ A	14.72 A	0.11 A	55.83 A	25.80 C
C × SD	*	*	*	*	*	*		*	*

* significant at *p* < 0.05. Data represent the mean and three replicates. Different letters are significantly different at *p* < 0.05 by least significant difference (LSD) test. GAE: gallic acid equivalent. FW: fresh weight.

**Table 2:** Analysis of variance (ANOVA) of KH Nps coating and storage durations on the gas concentration, respiration rate, RQ, AA, and TPC of papaya fruits.

Factor	Parameter	Mean Square	F-Value	Pr < F
Day	O_2_ Concentration	23.28	148.71	<0.001
	CO_2_ Concentration	2.17	262.79	<0.001
	C_2_H_4_ Concentration	66.83	62.44	<0.001
	O_2_ Rate	0.000069	9.60	0.0007
	CO_2_ Rate	0.000009	321.02	<0.001
	C_2_H_4_ Rate	44.29	44.67	<0.001
	RQ	0.007	256.95	<0.001
	AA	71.43	83.64	<0.001
	TPC	0.022	38.12	<0.001
Coating	O_2_ Concentration	30.28	193.44	<0.001
	CO_2_ Concentration	4.19	508.46	<0.001
	C_2_H_4_ Concentration	158.74	148.31	<0.001
	O_2_ Rate	0.000468	64.95	<0.001
	CO_2_ Rate	0.000014	462.00	<0.001
	C_2_H_4_ Rate	138.53	139.71	<0.001
	RQ	0.013	448.00	<0.001
	AA	100.49	117.66	<0.001
	TPC	0.003	6.19	0.0218
Day × Coating	O_2_ Concentration	20.97	6.98	<0.001
	CO_2_ Concentration	0.38	46.24	<0.001
	C_2_H_4_ Concentration	9.28	8.67	0.0012
	O_2_ Rate	0.000022	3.09	0.0569
	CO_2_ Rate	0.000002	51.90	<0.001
	C_2_H_4_ Rate	6.34	6.39	0.0047
	RQ	0.002	72.76	<0.001
	AA	16.84	19.72	<0.001
	TPC	0.002	3.15	0.0368

**Table 3:** Values of Peleg’s model parameter (*K*_1_, *K*_2_, and *R*^2^) for O_2_, CO_2_, and C_2_H_4_ concentrations in coated and control papaya.

Gas	Papaya	*K*_1_	*K*_2_	*R* ^2^
O_2_	Control	0.68 ± 0.35	25.15 ± 0.43	0.859
	Coated	2.35 ± 0.32	27.06 ± 0.27	0.997
CO_2_	Control	0.27 ± 0.59	0.29 ± 0.61	0.976
	Coated	0.66 ± 0.62	0.16 ± 0.54	0.997
C_2_H_4_	Control	3.53 ± 0.73	9.01 ± 0.55	0.998
	Coated	1.63 ± 0.77	8.59 ± 0.53	0.967

## References

[1] Singh S.P., Sudhakar Rao D.V. (2011). Papaya (Carica papaya L.).

[2] (2002). MOA Ministry of Agricultural and Agro-Based Industry. https://www.moa.gov.my/documents/20182/68591/Pelan+pemasaran+komoditi+betik.pdf/2db80b1b-04a4-42a0-ac53-02cb23345e2b.

[3] Mendy T.K., Misran A., Mahmud T.M.M., Ismail S.I. (2019). Application of Aloe vera coating delays ripening and extend the shelf life of papaya fruit. Sci. Hortic..

[4] Ho P.L., Tran D.T., Hertog M.L.A.T.M., Nicolaï B.M. (2020). Modelling respiration rate of dragon fruit as a function of gas composition and temperature. Sci. Hortic..

[5] Escamilla-García M., Rodríguez-Hernández M.J., Hernández-Hernández H.M., Delgado-Sánchez L.F., García-Almendárez B.E., Amaro-Reyes A., Regalado-González C. (2018). Effect of an edible coating based on chitosan and oxidized starch on shelf life of Carica papaya L., and its physicochemical and antimicrobial properties. Coatings.

[6] Adiletta G., Di Matteo M., Albanese D., Farina V., Cinquanta L., Corona O., Magri A., Petriccione M. (2020). Changes in physico-chemical traits and enzymes oxidative system during cold storage of “Formosa” papaya fresh cut fruits grown in the mediterranean area (Sicily). Ital. J. Food Sci..

[7] Islam M.Z., Saha T., Monalisa K., Hoque M.M. (2019). Effect of starch edible coating on drying characteristics and antioxidant properties of papaya. J. Food Meas. Charact..

[8] Nawab A., Alam F., Hasnain A. (2017). Mango kernel starch as a novel edible coating for enhancing shelf- life of tomato (*Solanum lycopersicum*) fruit. Int. J. Biol. Macromol..

[9] Zahedi S.M., Hosseini M.S., Karimi M., Ebrahimzadeh A. (2019). Effects of postharvest polyamine application and edible coating on maintaining quality of mango (*Mangifera indica* L.) cv. Langra during cold storage. Food Sci. Nutr..

[10] Mendoza R., Castellanos D.A., García J.C., Vargas J.C., Herrera A.O. (2016). Ethylene production, respiration and gas exchange modelling in modified atmosphere packaging for banana fruits. Int. J. Food Sci. Technol..

[11] Thakur R., Pristijono P., Bowyer M., Singh S.P., Scarlett C.J., Stathopoulos C.E., Vuong Q.V. (2019). A starch edible surface coating delays banana fruit ripening. LWT Food Sci. Technol..

[12] Maringgal B., Hashim N., Mohamed Amin Tawakkal I.S., Muda Mohamed M.T. (2020). Recent advance in edible coating and its effect on fresh/fresh-cut fruits quality. Trends Food Sci. Technol..

[13] Ali A., Muhammad M.T.M., Sijam K., Siddiqui Y. (2011). Effect of chitosan coatings on the physicochemical characteristics of Eksotika II papaya (*Carica papaya* L.) fruit during cold storage. Food Chem..

[14] Tabassum N., Khan M.A. (2020). Modified atmosphere packaging of fresh-cut papaya using alginate based edible coating: Quality evaluation and shelf life study. Sci. Hortic..

[15] Parven A., Sarker M.R., Megharaj M., Meftaul I.M. (2020). Prolonging the shelf life of Papaya (*Carica papaya* L.) using Aloe vera gel at ambient temperature. Sci. Hortic..

[16] Farina V., Passafiume R., Tinebra I., Scuderi D., Saletta F., Gugliuzza G., Gallotta A., Sortino G. (2020). Postharvest application of aloe vera gel-based edible coating to improve the quality and storage stability of fresh-cut papaya. J. Food Qual..

[17] Maringgal B., Hashim N., Tawakkal I.S.M.A., Mohamed M.T.M., Hamzah M.H., Ali M.M., Razak M.F.H.A. (2020). Kinetics of quality changes in papayas *(Carica papaya* L.) coated with Malaysian stingless bee honey. Sci. Hortic..

[18] Nicolaï B.M., Hertog M.L.A.T.M., Ho Q.T., Verlinden B.E., Verboven P., Yahia E.M. (2009). Gas exchange modeling. Modified and Controlled Atmosphere for Storage, Transportation, and Packaging of Horticultural Commodities.

[19] Fonseca S.C., Oliveira F.A.R., Brecht J.K. (2002). Modelling respiration rate of fresh fruits and vegetables for modified atmosphere packages: A review. J. Food Eng..

[20] Rahman E.A.A., Talib R.A., Aziz M.G., Yusof Y.A. (2013). Modelling the effect of temperature on respiration rate of fresh cut papaya (*Carica papaya* L.) fruits. Food Sci. Biotechnol..

[21] Mahajan P.V., Goswami T.K. (2001). Enzyme kinetics based modelling of respiration rate for apple. J. Agric. Eng. Res..

[22] Tappi S., Mauro M.A., Tylewicz U., Dellarosa N., Dalla Rosa M., Rocculi P. (2017). Effects of calcium lactate and ascorbic acid on osmotic dehydration kinetics and metabolic profile of apples. Food Bioprod. Process..

[23] Bhande S.D., Ravindra M.R., Goswami T.K. (2008). Respiration rate of banana fruit under aerobic conditions at different storage temperatures. J. Food Eng..

[24] Chaguri L., Sanchez M.S., Flammia V.P., Tadini C.C. (2017). Green Banana (*Musa cavendishii*) Osmotic Dehydration by Non-Caloric Solutions: Modeling, Physical-Chemical Properties, Color, and Texture. Food Bioprocess. Technol..

[25] Ravindra M.R., Goswami T.K. (2008). Modelling the respiration rate of green mature mango under aerobic conditions. Biosyst. Eng..

[26] Bialik M., Wiktor A., Latocha P., Gondek E. (2018). Mass transfer in osmotic dehydration of kiwiberry: Experimental and mathematical modelling studies. Molecules.

[27] Castelo Branco Melo N.F., de MendonçaSoares B.L., Marques Diniz K., Ferreira Leal C., Canto D., Flores M.A.P., Henrique da Costa Tavares-Filho J., Galembeck A., Montenegro Stamford T.L., Montenegro Stamford-Arnaud T. (2018). Effects of fungal chitosan nanoparticles as eco-friendly edible coatings on the quality of postharvest table grapes. Postharvest Biol. Technol..

[28] Divya M., Vaseeharan B., Abinaya M., Vijayakumar S., Govindarajan M., Alharbi N.S., Kadaikunnan S., Khaled J.M., Benelli G. (2018). Biopolymer gelatin-coated zinc oxide nanoparticles showed high antibacterial, antibiofilm and anti-angiogenic activity. J. Photochem. Photobiol. B Biol..

[29] Dhital R., Mora N.B., Watson D.G., Kohli P., Choudhary R. (2018). Efficacy of limonene nano coatings on post-harvest shelf life of strawberries. LWT Food Sci. Technol..

[30] Vieira A.C.F., de Matos Fonseca J., Menezes N.M.C., Monteiro A.R., Valencia G.A. (2020). Active coatings based on hydroxypropyl methylcellulose and silver nanoparticles to extend the papaya (*Carica papaya* L.) shelf life. Int. J. Biol. Macromol..

[31] Maringgal B., Hashim N., Tawakkal I.S.M.A., Hamzah M.H., Mohamed M.T.M. (2020). Biosynthesis of CaO nanoparticles using Trigona sp. Honey: Physicochemical characterization, antifungal activity, and cytotoxicity properties. J. Mater. Res. Technol..

[32] Wall M.M. (2006). Ascorbic acid, vitamin A, and mineral composition of banana (*Musa* sp.) and papaya (*Carica papaya*) cultivars grown in Hawaii. J. Food Compos. Anal..

[33] Abu Bakar M.F., Mohamed M., Rahmat A., Fry J. (2009). Phytochemicals and antioxidant activity of different parts of bambangan (Mangifera pajang) and tarap (*Artocarpus odoratissimus*). Food Chem..

[34] Xu D., Qin H., Ren D. (2018). Prolonged preservation of tangerine fruits using chitosan/montmorillonite composite coating. Postharvest Biol. Technol..

[35] Meindrawan B., Suyatma N.E., Wardana A.A., Pamela V.Y. (2018). Nanocomposite coating based on carrageenan and ZnO nanoparticles to maintain the storage quality of mango. Food Packag. Shelf Life.

[36] Moalemiyan M., Ramaswamy H.S., Maftoonazad N. (2012). Pectin-based edible coating for shelf-life extension of Ataulfo mango. J. Food Process. Eng..

[37] Ali A., Maqbool M., Alderson P.G., Zahid N. (2013). Effect of gum arabic as an edible coating on antioxidant capacity of tomato (*Solanum lycopersicum* L.) fruit during storage. Postharvest Biol. Technol..

[38] Hu H., Zhou H., Li P. (2019). Lacquer wax coating improves the sensory and quality attributes of kiwifruit during ambient storage. Sci. Hortic..

[39] Jongsri P., Wangsomboondee T., Rojsitthisak P., Seraypheap K. (2016). Effect of molecular weights of chitosan coating on postharvest quality and physicochemical characteristics of mango fruit. LWT Food Sci. Technol..

[40] Chen C., Peng X., Zeng R., Chen M., Wan C., Chen J. (2016). Ficus hirta fruits extract incorporated into an alginate-based edible coating for Nanfeng mandarin preservation. Sci. Hortic..

[41] Santos T.M., Souza Filho M.d.S.M., Silva E.d.O., Silveira M.R.S.d., Miranda M.R.A.d., Lopes M.M.A., Azeredo H.M.C. (2018). Enhancing storage stability of guava with tannic acid-crosslinked zein coatings. Food Chem..

[42] Sapper M., Palou L., Pérez-Gago M.B., Chiralt A. (2019). Antifungal Starch-Gellan Edible coatings with thyme essential oil for the postharvest preservation of apple and persimmon. Coatings.

[43] Beaudry R.M. (1993). Effect of carbon dioxide partial pressure on blueberry fruit respiration and respiratory quotient. Postharvest Biol. Technol..

[44] Fagundes C., Carciofi B.A.M., Monteiro A.R. (2013). Estimate of respiration rate and physicochemical changes of fresh-cut apples stored under different temperatures. Food Sci. Technol..

[45] Peleg M. (1988). An Empirical Model for the Prediction. J. Food Sci..

[46] Deng Z., Jung J., Simonsen J., Zhao Y. (2017). Cellulose nanomaterials emulsion coatings for controlling physiological activity, modifying surface morphology, and enhancing storability of postharvest bananas (*Musa acuminate*). Food Chem..

[47] Magwaza L.S., Mditshwa A., Tesfay S.Z., Opara U.L. (2017). An overview of preharvest factors affecting vitamin C content of citrus fruit. Sci. Hortic..

[48] Kathiresan S., Lasekan O. (2019). Effects of glycerol and stearic acid on the performance of chickpea starch-based coatings applied to fresh-cut papaya. CYTA J. Food.

[49] Lee S.K., Kader A.A. (2000). Preharvest and postharvest factors influencing vitamin C content of horticultural crops. Postharvest Biol. Technol..

[50] Wills R.B.H., Widjanarko S.B. (1995). Changes in physiology, composition and sensory characteristics of australian papaya during ripening. Aust. J. Exp. Agric..

[51] Maftoonazad N., Ramaswamy H.S. (2019). Application and Evaluation of a Pectin-Based Edible Coating Process for Quality Change Kinetics and Shelf-Life Extension of Lime Fruit (*Citrus aurantifolium*). Coatings.

[52] Madani B., Mirshekari A., Sofo A., Tengku Muda Mohamed M. (2016). Preharvest calcium applications improve postharvest quality of papaya fruits (*Carica papaya* L. cv. Eksotika II). J. Plant. Nutr..

[53] Shahidi F., Yeo J.D. (2018). Bioactivities of phenolics by focusing on suppression of chronic diseases: A review. Int. J. Mol. Sci..

[54] Ayón-Reyna L.E., González-Robles A., Rendón-Maldonado J.G., Báez-Flores M.E., López-López M.E., Vega-García M.O. (2017). Application of a hydrothermal-calcium chloride treatment to inhibit postharvest anthracnose development in papaya. Postharvest Biol. Technol..

[55] Ghasemnezhad M., Shiri M.A., Sanavi M. (2010). Effect of chitosan coatings on some quality indices of apricot (*Prunus armeniaca* L.) during cold storage. Casp. J. Environ. Sci..

